# First Report on the Latvian SARS-CoV-2 Isolate Genetic Diversity

**DOI:** 10.3389/fmed.2021.626000

**Published:** 2021-04-06

**Authors:** Nikita Zrelovs, Monta Ustinova, Ivars Silamikelis, Liga Birzniece, Kaspars Megnis, Vita Rovite, Lauma Freimane, Laila Silamikele, Laura Ansone, Janis Pjalkovskis, Davids Fridmanis, Baiba Vilne, Marta Priedite, Anastasija Caica, Mikus Gavars, Dmitry Perminov, Jelena Storozenko, Oksana Savicka, Elina Dimina, Uga Dumpis, Janis Klovins

**Affiliations:** ^1^Latvian Biomedical Research and Study Centre, Riga, Latvia; ^2^Riga Stradins University, Riga, Latvia; ^3^Centrala Laboratorija, Ltd, Riga, Latvia; ^4^E. Gulbja Laboratorija, Ltd, Riga, Latvia; ^5^Faculty of Biology, University of Latvia, Riga, Latvia; ^6^Laboratory Service, Latvian Centre of Infectious Diseases Laboratory, National Microbiology Reference Laboratory, Molecular Biology and Virology Department, Riga East University Hospital, Riga, Latvia; ^7^Infectious Diseases Surveillance and Immunization Division, Infectious Diseases Risk Analysis and Prevention Department, The Centre for Disease Prevention and Control (CDPC) of Latvia, Riga, Latvia; ^8^Faculty of Medicine, University of Latvia, Riga, Latvia; ^9^Pauls Stradins Clinical University Hospital, Riga, Latvia

**Keywords:** Latvia, COVID-19, next-generation sequencing, genetic diversity, 2019-nCoV, HCoV-19, SARS-CoV-2

## Abstract

Remaining a major healthcare concern with nearly 29 million confirmed cases worldwide at the time of writing, novel severe acute respiratory syndrome coronavirus-2 (SARS-CoV-2) has caused more than 920 thousand deaths since its outbreak in China, December 2019. First case of a person testing positive for SARS-CoV-2 infection within the territory of the Republic of Latvia was registered on 2nd of March 2020, 9 days prior to the pandemic declaration by WHO. Since then, more than 277,000 tests were carried out confirming a total of 1,464 cases of coronavirus disease 2019 (COVID-19) in the country as of 12th of September 2020. Rapidly reacting to the spread of the infection, an ongoing sequencing campaign was started mid-March in collaboration with the local testing laboratories, with an ultimate goal in sequencing as much local viral isolates as possible, resulting in first full-length SARS-CoV-2 isolate genome sequences from the Baltics region being made publicly available in early April. With 133 viral isolates representing ~9.1% of the total COVID-19 cases during the “first coronavirus wave” in the country (early March, 2020—mid-September, 2020) being completely sequenced as of today, here, we provide a first report on the genetic diversity of Latvian SARS-CoV-2 isolates.

## Introduction

Current novel coronavirus disease (COVID-19) pandemic caused by severe acute respiratory syndrome coronavirus 2 (SARS-CoV-2), which was formerly known as 2019 novel coronavirus (2019-nCoV), and is often referred to as human coronavirus 2019 (hCoV-19), responsible for a sudden rise in pneumonia cases in Wuhan, China, late December 2019, was preventively deemed a Public Health Emergency of International Concern by WHO as early as 30th January, 2020 with only as few as 7,836 cases confirmed worldwide back then. With rapidly growing number of confirmed positive cases throughout the world, SARS-CoV-2 quickly became arguably the most sequenced virus in history with more than 100 thousand (14 September 2020) viral isolate near complete genome sequences of high quality available publicly at the time of writing at GISAID repository thanks to the unprecedented rate of collaborations between researchers and unpublished data sharing with the goal of effectively tackling the novel disease (1, 2).

### Genome of SARS-CoV-2

First reported genomic sequence of SARS-CoV-2 was deduced from a metagenomic RNA of bronchoalveolar lavage fluid specimen sampled from a patient who worked at Wuhan seafood market, where the epidemiological onset of human-to-human transmission of a novel zoonotic coronavirus is thought to have taken place (3), although evidence of an earlier contraction of the disease that was not associated with the seafood market has been documented, leading to the conclusion that the primary spill-over event has taken place elsewhere (4, 5). The sequence of a 29,903 base-long non-segmented positive-sense single-stranded RNA molecule representing complete genome of the aforementioned isolate Wuhan-Hu-1 was deposited in GenBank (6) on 5th of January, 2020 and is now known as a SARS-CoV-2 reference sequence available under accession numbers NC_045512.2 or MN908947.3.

While viral family *Coronaviridae*, that comprises α/β/Δ/γ coronavirus genera, representatives are somewhat unique in comparison with most other RNA viruses in regards to their large genome size of ~30 kb, genomic organization of individual species does not differ much among other lower taxa within the family, while boasting variable number of open reading frames. The genome of SARS-CoV-2 begins with a 265-base-long 5′-UTR region starting with a leader sequence followed by a 21,290-base-long ORF1ab, comprising about 70% of the genome length, that translates into two polyproteins *via* −1 ribosomal frameshift and encodes 16 non-structural proteins (nsp1–nsp16). The remaining part of the genome comprises ORFs coding for structural and accessory proteins of unknown function, sequentially: Spike glycoprotein (S), ORF3a, envelope protein (E), membrane glycoprotein (M), ORF6, ORF7a, ORF7b, ORF8, nucleocapsid phosphoprotein (N), ORF10, followed by 3′-UTR ending in poly(A) tail. However, no evidence that would support the expression of SARS-CoV-2 ORF10-encoded protein of unknown function is yet found in the literature (7).

### Possible Origins of SARS-CoV-2

SARS-CoV-2 is the seventh zoonotic human coronavirus known up to date, and, along with SARS-CoV and MERS-CoV, is considered to be highly pathogenic and more severe compared with other, milder symptoms causing, community-acquired human coronaviruses (HCoV-229E, HCoV-OC43, HCoV-HKU1, and HCoV-NL63) (8).

Studies on the origin of novel coronavirus have revealed that complete genomic sequence of SARS-CoV-2 suggests a more close, although not a direct parental, ancestral relationship with bat [~96% overall nucleotide homology with RaTG13 (9)] and pangolin coronaviruses [up to ~92% homology, with S protein ACE2 receptor binding domain amino acid sequence being 97.4% identical to SARS-CoV-2 (10)], than to those of humans (~79 and ~50% identity to SARS-CoV and MERS-CoV, respectively (11)), and, while bats are already a long-time acknowledged reservoir of SARS-CoV-like β-coronaviruses (12, 13), the assumption that pangolins can serve as a natural host for CoVs has been made only recently before the emergence of SARS-CoV-2 (14, 15). Although the current risk of animal-human transmission of COVID-19 is considered low, a number of felines (16), canines (17), and minks (18) worldwide have been reported to be infected with SARS-CoV-2.

### SARS-CoV-2 Isolate Classification

With a steadily growing number of complete SARS-CoV-2 genome sequences, early efforts to classify novel isolates based on their genetic make-up have resulted in numerous proposals of different SARS-CoV-2 isolate classification systems (19–21), some of which (e.g., PANGOLIN lineages) are complementary. However, with more than 100,000 of SARS-CoV-2 genome sequences being available publicly as of now, ongoing efforts to aid in the classification of newly sequenced viral isolates have resulted in the general acceptance of GISAID's team developed SARS-CoV-2 major clade and lineage nomenclature system based on the specific combinations of 9 SARS-CoV-2 genetic markers (2). In accordance with this system, SARS-CoV-2 isolates can be classified in at least six distinct major clades, namely: S, L (containing reference sequence Wuhan-Hu-1), V, G, GH, GR, and O (other) isolate clades (Table 1).

**Table 1:** Major SARS-CoV-2 clades defining genetic markers and their occurrence in Latvia, Europe, and Worldwide (as of 14 September 2020).

**Clade**	**genetic markers**									**(*n* = 100272)**	**(*n* = 52641)**	
	**241 bp**	**3037 bp**	**23403 bp**	**8782 bp**	**11083 bp**	**25563 bp**	**26144 bp**	**28144 bp**	**28882 bp**	**Percent of isolates Worldwide (%)**	**Percent of isolates in Europe (%)**	**Percent of isolates in Latvia (%)**
S	C	C	A	T	G	G	G	C	G	5.79	2.82	0
L	C	C	A	C	G	G	G	T	G	4.31	5.78	3.01
V	C	C	A	C	T	G	T	T	G	5.16	8.46	0
G	T	T	G	C	G	G	G	T	G	22.59	28.54	16.54
GH	T	T	G	C	G	T	G	T	G	22.14	10.56	30.83
GR	T	T	G	C	G	G	G	T	A	34.92	41.79	48.12
Other	X	X	X	X	X	X	X	X	X	5.09	2.05	1.5

*X denotes any nucleotide*.

Mid-September, 2020, the most represented clades Worldwide are GR, G, and GH, roughly corresponding to 34.92, 22.59, and 22.14% of total SARS-CoV-2 isolates, respectively. All three of these clades are characterized by C241T base substitution in 5′-UTR region, C3037T silent mutation in ORF1a and missense A23403G mutation that causes aspartic acid at position 614 of spike glycoprotein (S) to change to glycine (S-D614G), that is associated with higher viral loads and, in turn, is hypothesized to increase the infectivity of these genotypes, with isolates bearing this mutation quickly becoming dominant ones in various regions throughout the world (22–24). More recent clades GR and GH are further distinguished from the ancestral G genotype by G25563T mutation resulting in position 57 of ORF3a protein to change from glutamine to histidine for clade GH, and G28882A that changes glycine at nucleocapsid phosphoprotein (N) aa position 204 to arginine for clade GR. While the exact effect of GH clade-defining G25563T change in apoptosis-inducing transmembrane ORF3a protein (Q57R) remains unknown, it does not seem to affect any of the conserved functional domains distinguishable within the protein (25, 26). Whereas, G28882A mutation associated with GR genotype is almost always a trinucleotide mutation of neighboring loci resulting in GGG to AAC change at positions 28881, 28882, and 28883, respectively. This trinucleotide mutation results in two (R203K and G204R) consecutive amino acid changes in N protein, which, in turn, might have potential implications on nucleocapsid phosphoprotein structure and/or function via reduction of conformational entropy and changes in inter-residue interactions in the proximity of the mutated amino acid positions [elaborated on in (27)]. The currently estimated evolutionary rate of SARS-CoV-2 is around 9.86 × 10^−4^ to 1.85 × 10^−3^ substitutions per position per year (28), and, based on the isolates sequenced worldwide up to date, there is evidence that mutations in nearly every position in the genome of SARS-CoV-2 have already been documented (29).

In this study, we are reporting the first results of an ongoing massive sequencing campaign that allows us to elaborate on the genetic diversity of SARS-CoV-2 isolates from Latvian patients.

## Materials and Methods

### Sample Management and Detection of SARS-CoV-2

For viral genome analysis, either oropharyngeal or nasopharyngeal swabs obtained from COVID-19 patients or already extracted RNA samples were provided to Latvian Biomedical Research and Study Center by the three accredited diagnostic laboratories (E. Gulbis Laboratory, Central Laboratory and Latvian Center of Infectious Diseases) covering diagnostics of all officially reported cases of SARS-CoV-2. RNA extraction from oropharyngeal and nasopharyngeal swabs and the following SARS-CoV-2 detection was performed by multiple different methods according to standard procedures of each laboratory. These included manual Trizol-based RNA extraction (TRI reagent, Sigma) and automated purification methods with STARMag 96 X 4 Universal Cartridge Kit (Seegene Inc.), NucliSENS easyMAG (bioMérieux), QIAamp 96 Virus QIAcube HT Kit (QIAGEN). The presence of SARS-CoV-2 in the purified RNA samples for the diagnostics was estimated by, either commercial (Allplex™ 2019-nCoV Assay, Seegene Inc, detecting the E, RdRp and N genes according to manufacturer instructions) or in-house RT-qPCR methods (detecting the N and S genes) (30), or even both to ensure the technical validation of the obtained test results. Samples showing amplification (ct <40) of at least one viral gene (RdRp, E, N) were considered positive and directed to next-generation sequencing.

### Next-Generation Sequencing Approach Selection

Metatranscriptome sequencing was the first-choice method for the SARS-CoV-2 genome analysis. Nevertheless, since the majority of samples showed an insufficient number of sequencing reads mapping to the SARS-CoV-2 genome and could not be reliably analyzed, targeted sequencing approaches were considered. A methodological strategy plan was developed in order to apply the most effective next-generation sequencing method for each sample according to the quantity of SARS-CoV-2 (Supplementary Figure 1). At first, RT-qPCR was repeated for each sample in order to evaluate the quantity of viral RNA with a common approach for all samples. Three SARS-CoV-2 genome-specific primer pairs and probes targeting different regions of the nucleocapsid protein (N) gene implemented in the 2019-nCoV RUO Kit (IDT) and SOLIScript^®^ 1-step CoV Kit (Solis Biodyne) were used for the amplification (Supplementary Table 3). Probes N1 and N2 specifically detected SARS-CoV-2, while the N3 probe universally detected all currently recognized clade 2 and 3 viruses within the subgenus Sarbecovirus (31). To evaluate the RNA extraction and PCR efficiency, simultaneous amplification of the human RNase P gene was performed and a control sample containing a plasmid with the SARS-CoV-2 nucleoplasmid protein gene (2019-nCoV_N_Positive Control, IDT) was added to each reaction set. The potential contamination was evaluated by a negative control (nuclease-free water instead of RNA) added to each sample set. RT-qPCR was conducted on the ViiA 7 Real-Time PCR System (Thermo Fisher Scientific), and only the samples showing amplification (ct <40) of all three SARS-CoV-2 nucleoplasmid protein genes were further directed to metatranscriptome sequencing. Samples exhibiting poor amplification of viral genes (ct >40 for at least one target region) were considered for one of targeted sequencing approaches: hybridization capture or amplification of SARS-CoV-2.

#### Metatranscriptome Sequencing

In order to eliminate contaminating DNA, DNase I treatment (NEB) of RNA samples was performed, followed by rRNA depletion with MGIEasy rRNA Depletion Kit (MGI Tech Co. Ltd). Complementary DNA libraries were prepared using MGIEasy RNA Library Prep Set (MGI Tech Co. Ltd). Quantity and quality of both RNA and cDNA were evaluated using the Qubit 2.0 fluorometer and Agilent 2100 Bioanalyzer system, respectively. The presence of the SARS-CoV-2 genome was repeatedly tested in each cDNA library by Q-PCR before sequencing, using the same primers and probes (2019-nCoV_N_Positive Control, IDT) together with TaqMan™ Gene Expression Master Mix (Thermo Fisher Scientific). After multiple experimental tests, a ct value threshold of 25 was chosen for N1 and N3 probes for cDNA libraries to be forwarded to metatranscriptome sequencing (N2 probe appeared to be unstable and therefore uninformative). Metatranscriptome cDNA libraries were sequenced on the DNBSEQ-G400RS sequencing platform with DNBSEQ-G400RS High-throughput Sequencing Set (PE150) (MGI Tech Co. Ltd), obtaining at least 100 million 150-bp-paired-end sequencing reads per each sample. Those libraries that failed to pass the ct threshold (ct >25 for N1 and N3) were directed to a targeted approach.

#### SARS-CoV-2 Hybridization Capture

One of the targeted sequencing strategies was based on the enrichment of the SARS-CoV-2 genome by hybridization probes. For cDNA library preparation TruSeq Stranded Total RNA Library Prep Gold kit and TruSeq RNA UD Indexes (Illumina) were used. The indexed cDNA libraries were enriched for the SARS-CoV-2 genome using compatible hybridization probes implemented in the myBaits Expert SARS-CoV-2 kit (Arbor Biosciences) according to manufacturers' instructions. See the official webpage of the manufacturer (https://arborbiosci.com/) for the full list of hybridization probes used. The enriched libraries were sequenced on Illumina MiSeq system with MiSeq Reagent Kit v3 (150-cycle), obtaining at least 1 million of around 75-bp-paired-end reads per sample.

#### Amplification of SARS-CoV-2 Genome

The second targeted approach involved multiplexed primers for amplification of the whole SARS-CoV-2 genome. QIAseq SARS-CoV-2 Primer Panel (QIAGEN) based on the study from the ARTIC network [https://github.com/artic-network/artic-ncov2019, (32)] was used together with QIAseq FX DNA Library Kit (QIAGEN) for cDNA library preparation. Next-generation sequencing was performed on Illumina MiSeq system with MiSeq Reagent Kit v2 (300-cycles), obtaining at least 1 million of around 150bp paired-end reads per sample.

### Sequencing Data Quality Control, Variant Calling, and Data Sharing

Adapter clipping was performed with cutadapt 1.16 (33). Subsequent read trimming was performed with fastp 0.20.0 (34) using five base-sliding window trimming from both ends with quality threshold 20. Reads with length <75 bp or an average quality of <20 were removed. Quality-controlled reads were then aligned against SARS-CoV-2 isolate Wuhan-Hu-1 reference genome (Accession number: NC_045512.2) with bowtie2 2.3.5.1 (35). Variant calling and consensus sequence construction were implemented using bcftools 1.10.2 (36). Average coverage for each of the genomes was calculated using samtools and in-house awk (37, 38) scripts. Less than 1% of the missing bases were allowed for a genome to be considered successfully sequenced and missing bases were treated as reference bases from the Wuhan-Hu-1 genome. Consensus sequences of the successfully sequenced isolates were then proceeded to the manual variant quality inspection by sequence alignment map visualization in IGV (39), sequences that have passed the manual variant quality check were immediately publicly shared by deposition to GISAID database (2). Variant annotations were performed using coronapp SARS-CoV-2 genome autoannotation web server by comparisons to reference sequence (40) and the results were summarized with the help of custom R scripts, ggplot2 R library was used for plot visualizations (41, 42).

### Phylogenetic Reconstructions

The dataset (alignment of 133 Latvian SARS-CoV-2 isolates and Wuhan-Hu-1 reference sequence) was tested for the presence of a phylogenetic signal prior to our phylogenetic analyses by the likelihood mapping analysis implemented in IQTREE version 2.0.6 (using 1,000× number of samples (134,000) randomly drawn quartets) (Supplementary Figure 4) (43, 44). Sequences of the Latvian SARS-CoV-2 isolates and Wuhan-Hu-1 reference sequence were aligned using Clustal-Omega v1.2.4 (45). Maximum likelihood phylogeny was performed using IQTREE v2.0.6 (44) with GTR+F+I as best fit model determined by ModelFinder (46) according to Bayesian Information Criterion [ultrafast bootstrap with 1,000 replicates (47)] and assessment of temporal signal associated with the data was performed by importing resulting ML tree into TempEst v1.5.3 (48), parsing sampling dates of isolates and visualizing the root-to-tip divergence.

Bayesian phylogenetic trees were estimated using BEAST v1.10.4 (49), employing GTR nucleotide substitution model with empirical base frequencies and invariant site proportion assuming strict molecular clock. Coalescent exponential growth prior (growth rate prior: Laplace with scale 100; population size prior: Lognormal with mu 1 and sigma 2) with growth rate parametrization (50, 51) was selected and Markov chain Monte Carlo (MCMC) was run for 50 million states sampling log parameters and trees every 5,000 states. Tracer v1.7.1 (52) was used for MCMC trace (log file) inspection to evaluate sufficiency of sampling (all parameters had an ESS of more than 400) and infer substitution rate along with the date of the most recent common ancestor estimate. To summarize Bayesian phylogenetic inference, maximum clade credibility time-scaled tree was generated in TreeAnnotator v1.10.4 (distributed with BEAST package) using 10% of the states (5 million) as the burn-in and visualized using FigTree v1.4.4 (53).

## Results and Discussion

**Figure 1:**
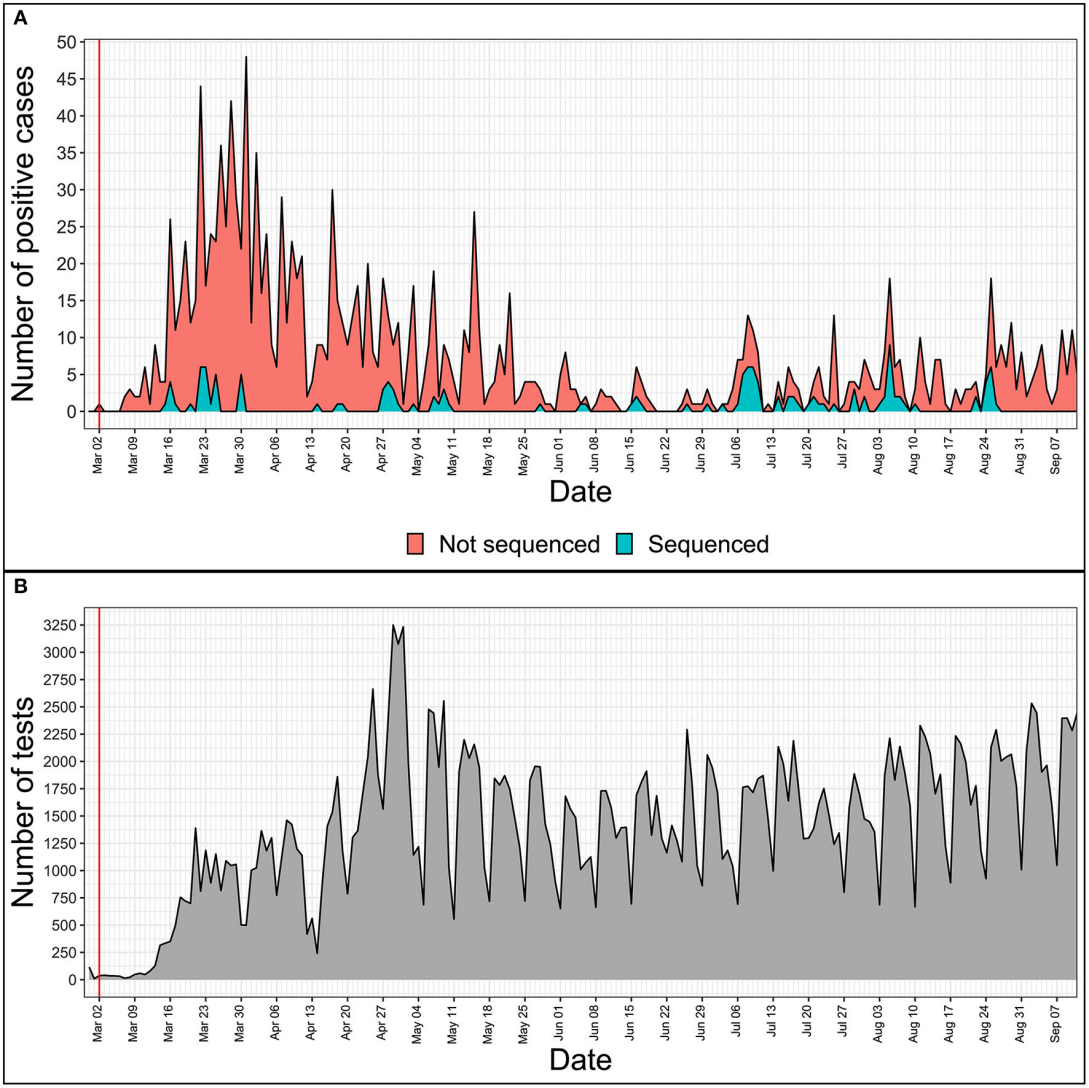
Daily numbers of positive COVID-19 cases **(A)** and tests performed **(B)** in Latvia. *x*-axis is the same for both tiles and represents daily time series from 28th of February, 2020 to 11th of September, 2020. The red vertical line indicates the date of the first COVID-19 case registered in Latvia. **(A)** *Y* value represents the total number of positive cases registered on a given day. Blue area shows the number of only successfully sequenced isolates, while the red area represents the positive cases not sequenced during this study. **(B)** *Y* value represents the number of tests carried out on a given date in Latvia.

With 1,464 cumulative positive cases as of 12th of September 2020 (1,248 people recovered, 181 active cases of the disease, and 35 COVID-19-associated deaths), 133 SARS-CoV-2 isolates representing ~9.1% of the total local COVID-19 cases have been completely sequenced as of today, making Latvia one of the leading countries not only in regards to the containment of the spread of COVID-19 disease, but in the number of sequenced SARS-CoV-2 isolates to the cumulative number of positive COVID-19 cases ratio as well (Figure 1).

Reacting to the emergence of the SARS-CoV-2 in Latvia, a high-throughput framework for SARS-CoV-2 isolate sequencing and data analysis with capabilities of near real-time tracking of the epidemiological situation in Latvia was built to aid the governmental decision-making and study the molecular epidemiology of hCoV-19.

One of the challenges to obtain good-quality sequences for maximal number of samples is the variable quality of input material that can be caused by highly variable viral loads, different collection, storage and RNA isolation methods. Although for the current study we did not have the information on the severity of COVID-19 symptoms for particular cases, it should be noted that the absolute majority of cases in Latvia are with low symptom severity expected to have lower concentration of virus in diagnostic samples. We therefore developed an approach to verify sample quality and select appropriate sequencing method to recover maximal available information from existing samples ensuring cost efficacy of the process (Supplementary Figure 1). According to this strategy developed during the implementation of the study, complete SARS-CoV-2 genome sequence was successfully obtained by metatranscriptome approach for 37 viral isolates, 80 samples were analyzed by amplification of SARS-CoV-2 genome with multiplexed primers, and for 16 isolates enrichment of SARS-CoV-2 genome was performed by hybridization capture method prior the sequencing.

As of now, it could be cautiously speculated that the obtained results on the SARS-CoV-2 genotype distributions might be somewhat representative of a whole Baltics region, taking the geographical proximity, travel habits, and mild governmental travel regulations between the Baltic states during the most of the pandemic into the account. However, the extent of similarity between the isolates circulating in different Baltic states currently cannot be reliably established due to SARS-CoV-2 isolate undersequencing in neighboring Estonia and Lithuania, and the founder effect of multiple independent (re-)introductions of different SARS-CoV-2 genotypes, as well as containment effectivity of respective COVID-19 cases, in each of the countries should not be overlooked.

### Distribution of Sequenced Virus Isolates by SARS-CoV-2 Clades

**Figure 2:**
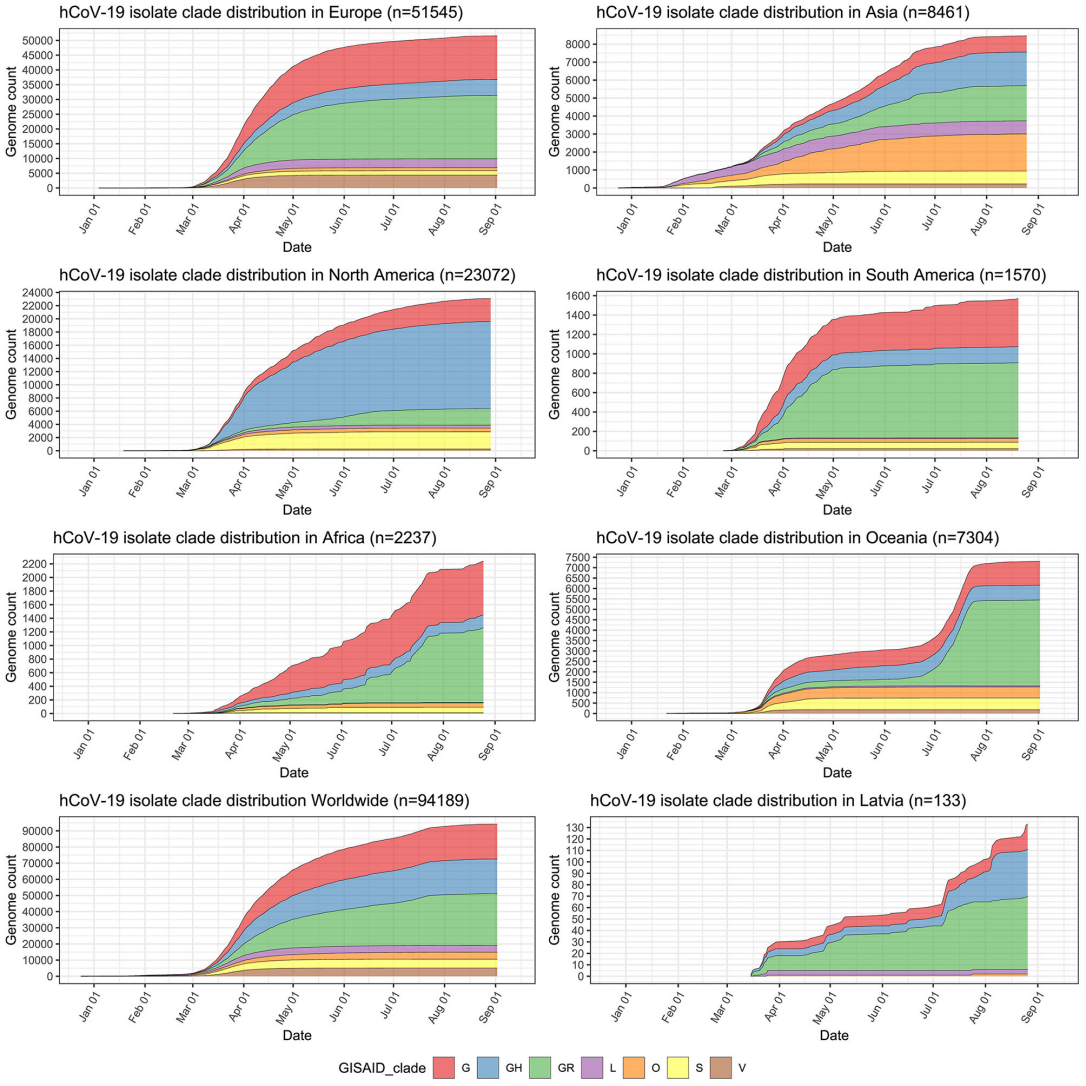
Distribution of sequenced SARS-CoV-2 isolates by clades in major regions of the world, worldwide, and in Latvia. *y*-axis depicts cumulative complete SARS-CoV-2 genome count (with unambiguous collection date) from a particular region and has different scale within the subplots. *x*-axis is the same for all subplots and depicts sampling time-series from 24th of December, 2019 till 12th of September, 2020.

Major isolate clade distributions across distinct geographical regions show clear spatial differences of the epidemic (Figure 2) and a trend of “older” isolate clades L and S losing their initial prevalence to the dominance of the more recently emerged G-associated clades (G, GH, GR) that seem to be accountable for the majority of the cases worldwide since the middle of March 2020. GR, which is the most common isolate clade in Latvia (48.12% of cases), is also a dominant clade in Europe and South America. Currently, GH still seems to be the most common isolate clade circulating throughout North America, but a rise in the number of GR isolates can be observed since the middle of May 2020. The prevalence of GR and, in particular, G clade isolates is also currently on the rise in Africa, and, to a very moderate amount in Oceania and Asia. The relatively high number of isolates not corresponding to any of the currently recognized major SARS-CoV-2 clades (dubbed “Other” or belonging to the “O” clade as of now) in Asia and Oceania makes it possible to speculate about it being indicative of either (but not mutually exclusive), poor quality of the sequences obtained or the possibility of novel clade emergence originating from these regions in the future, should their spread not be effectively contained.

### Mutational Landscape of Latvian SARS-CoV-2 Isolates

After joining of the neighboring loci, among 133 local isolates, 247 different unique mutational events (154 non-synonymous, 84 synonymous, 7 substitutions in extragenic regions, single deletion, and a single stop codon introduction) that affected 244 positions of the SARS-CoV-2 genome were registered from a total of 1,355 variants that were identified. One hundred forty-six out of 247 distinct mutational events were registered only in one of the 133 samples, while 101 were present in two or more samples (Supplementary Table 1; Supplementary Figure 2). NSP3 was found to be the mature peptide most frequently affected by non-synonymous substitutions (24 distinct variants resulting in an amino acid change), followed by an N protein that had 15 non-synonymous SNVs documented among Latvian SARS-CoV-2 isolates. Among the most frequently mutated proteins, NSP2, S, and NSP12b mature peptides harbored 13, 12, and 10 different amino acid altering mutations, respectively.

Based on the current coronapp web-server [38] report updated at 15 September 2020 (*n* = 89,978), most frequent mutational events worldwide are as follows: A23403G corresponding to S:D614G, C3037T silent mutation, C14408T resulting in NSP12b:P314L, C241T extragenic substitution and GGG28881ACC trinucleotide mutation of neighboring loci resulting in N:RG203KR, G25563T—ORF3a:Q57H. All six of these mutations were also among the most frequent mutational events registered in Latvian samples: 5′-UTR C241T extragenic substitution that was present in 129 out of 133 sequenced genomes, while C3037T silent (NSP3:F106F) mutation, A23403G (S:D614G), and C14408T (NSP12b:P314L) were all present in 128/133 samples, GGG28881AAC trinucleotide mutation (N:RG203KR) was observed in 59/133 of the samples, and 28881 position of the genome had two more variants detectable in the samples—GGGG28881AACT (N:RG203KL) quadranucleotide mutation (32/133) and G28881A (N:R203K) substitution being present in five of the samples, while G25563T—ORF3a:Q57H was found in 41 of the isolates (see Table 2; Supplementary Table 1).

**Table 2:** Ten most frequently mutated genome positions among Latvian SARS-CoV-2 isolates (*n* = 133).

**Position in genome**	**Reference**	**Variant**	**Variant**	**Variant class**	**Region affected**	**Amino acid change**	**Function of the mature peptide**	**Variant**		**Occurrence among**	
								**frequency**		**Latvian isolates**	
241	C	5′UTR:241	T	Extragenic	5′UTR	241	N/A	129		96.99%	
3,037	C	NSP3:F106F	T	Silent	NSP3	F106F	Predicted phosphoesterase, papain-like proteinase	128		96.24%	
14,408	C	NSP12b:P314L	T	SNP	NSP12b	P314L	RNA-dependent RNA polymerase, post-ribosomal frameshift	128		96.24%	
23,403	A	S:D614G	G	SNP	S	D614G	Spike	128		96.24%	
	GGG	N:RG203KR	AAC			RG203KR		59		44.36%	
28,881	G	N:R203K	A	SNP^*^	N	R203K	Nucleocapsid protein	32	96	24.06%	72.18%
	GGGG	N:RG203KL	AACT			RG203KL		5		3.76%	
25,563	G	ORF3a:Q57H	T	SNP	ORF3a	Q57H	ORF3a protein	41		30.83%	
18,877	C	NSP14:C279C	T	Silent	NSP14	C279C	3′-to-5′ exonuclease	36		27.07%	
1,202	A	NSP2:N133D	G	SNP	NSP2	N133D	Non-Structural protein 2	34		25.56%	
12,513	C	NSP8:T141M	T	SNP	NSP8	T141M	Non-Structural Protein 8	34		25.56%	
25,710	C	ORF3a:L106L	T	Silent	ORF3a	L106L	ORF3a protein	33		24.81%	

Color coding is based on the variant class, as follows: red represents extragenic variants; green, silent variants; and blue, single nucleotide polymorphisms. Asterisk

(*) *in “Variant class” column represents that there are multiple variants present at a given genome position (28,881); some of them are neighboring loci polynucleotide variants rather than SNP*.

It was noted that five out of six aforementioned mutations (with the exclusion of C14408T) are in the genome positions serving as markers for current SARS-CoV-2 isolate major clade definition and correspond to GR clade, that is the most represented clade Worldwide and hosts almost half of the sequenced isolates in Latvia (Tables 1, 2). The C14408T substitution resulting in NSP12b:P314L amino acid change has been previously reported to co-occur with C241T, C3037T, and A23403G mutations (54), which is consistent with our data, where four of these SNPs were simultaneously present 127/133 of the Latvian SARS-CoV-2 isolates sequenced up to date. While no experimental evidence of C14408T substitution implications on the NSP12b (RdRp) activity is yet present, isolates bearing this variant were previously speculated to have more mutations, and elaborations about possible implications of RdRp mutations on antiviral drug resistance were made (55). The fitness of G and G-derived strains, as denoted by the recent rise of their prevalence throughout different regions of the world, is hardly explainable only by the founder effect alone, thus highlighting the fact that further evidence on molecular and clinical implications of the most common substitutions in the genomes of currently circulating SARS-CoV-2 is urgently needed to improve the measures of containment of COVID-19 and develop effective antiviral therapies and vaccines, that would help to not only combat the present virus of immediate concern but also be of vital importance for other coronaviruses to yet emerge.

### Phylogenetic Analyses

Likelihood mapping analysis conducted to evaluate the presence of phylogenetic signal suggested that there, indeed, is a phylogenetic signal in our dataset (Supplementary Figure 4, <1/3 of the quartets unresolved, 64.68% of quartets fully resolved). Root-to-tip regression analysis with the “best-fitting root” and “correlation” function options resulted in a correlation coefficient of the analysis being estimated at 0.6754 and a determination coefficient (*R*^2^) equaling to 0.4562 (Supplementary Figure 3). Although having some of the sequences that diverged more or less than expected at their sampling date, the dataset had a moderate association between sequence divergence and sampling date, implying suitability for phylogenetic molecular clock analysis.

**Figure 3:**
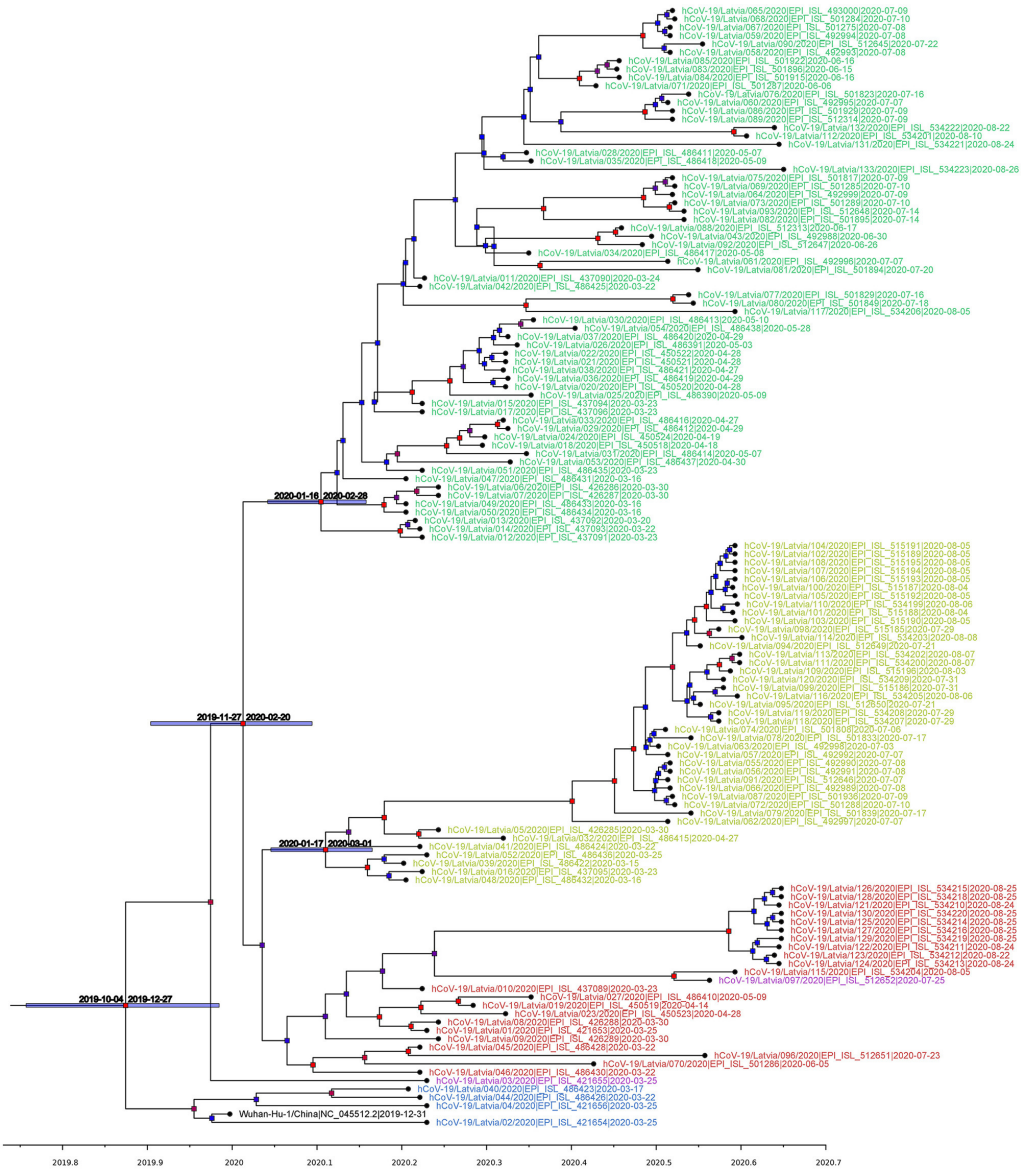
Maximum clade credibility tree (mean node heights) estimated from the completely sequenced Latvian isolates (*n* = 133) and Wuhan-Hu-1 isolate. Node labels are colored according to the GISAID major clade of particular isolate, as follows: green, GR; yellow, GH; red, G; blue, L; purple, O (other); black, Wuhan-Hu-1 reference sequence. The tree is time scaled and axis represents time in a decimal year notation (1 months is ~0.08333 of a year and 1 day is ~0.00274 of a year). Nodes are colored according to their respective posterior probabilities in gradient from blue (lowest value) to red (highest value). Dated node bars represent 95% highest posterior density intervals and are shown for the selected nodes.

Following Bayesian phylogenetic inference, mean evolutionary rate derived from Latvian SARS-CoV-2 isolates was found to be 7.5185 × 10^−4^ substitutions per site per year (6.0256 × 10^−4^-9.1308 × 10^−4^, 95% highest posterior density interval), roughly corresponding to an average of 22–23 mutational events in genome per year (95% HPD: ~18 to ~27), and lies within or close to the evolutionary rate ranges predicted by other researchers (56–59). Based on the analysis, the estimated most recent common ancestor of the isolates has emerged on 16th of November, 2019 (4th October, 2019–27th December, 2019, 95% interval). Our molecular clock analysis (Figure 3) further supported the more recent divergence of G and G-derived (GR and GH) clades with the most recent common ancestor for three of the aforementioned major clades dating back to 6th of January, 2020 (95% HPD: 27th November, 2019–5th February, 2020) and allowed us to date the near-simultaneous emergence of TMRCAs for clades GR (8th of February, 2020; 95% HPD: 16th January, 2020–28th of February, 2020) and GH (10th of February, 2020; 95% HPD: 17th January, 2020–1st March, 2020). The 95% HPD date ranges are consistent with the collection dates of unambiguously dated genomes belonging to clades GH and GR deposited at GISAID (accessed 14 August 2020). Earliest reported SARS-CoV-2 genome belonging to clade GH was collected on 2nd of February 2020 in Riyadh, Saudi Arabia (GISAID accession: EPI_ISL_489996), while earliest reported GR clade genome was collected on 16th of February 2020 in London, England (GISAID accession: EPI_ISL_466615), however first reported sequences with unambiguous collection date belonging to GR and GH ancestral clade G were collected on 24th of January, 2020, in China, cities of Zhejiang and Chengdu (GISAID accessions: EPI_ISL_422425, EPI_ISL_451345).

Our phylogenetic analysis of the local isolates suggests multiple unlinked initial introductions of already divergent SARS-CoV-2 isolates to Latvia. Just 2 weeks after the first positive case of COVID-19 was documented in Latvia on the 2nd of March, isolates representing at least three major SARS-CoV-2 clades (L, GR, and GH) were already circulating within the country corresponding to at least four epidemiologically unlinked introductions. No isolates belonging to clade L (most similar to the initial Wuhan-Hu-1 reference) were sequenced after the end of March and local circulation of clade G representatives was not detectable until the end of August, while clade GH and, specifically, GR isolates seem to have taken hold of the epidemic without showing any signs of ceasing their proliferation within the Latvian population; however, recent reintroduction event possibility should not be ruled out due to cancelation of travel restrictions and insufficient testing of those entering the country. With nearly half of the sequenced isolates belonging to the widely represented GR clade, up to this date, no isolates representing clades V or S were documented among the sequenced Latvian COVID-19 cases (Figures 2, 3).

**Figure 4:**
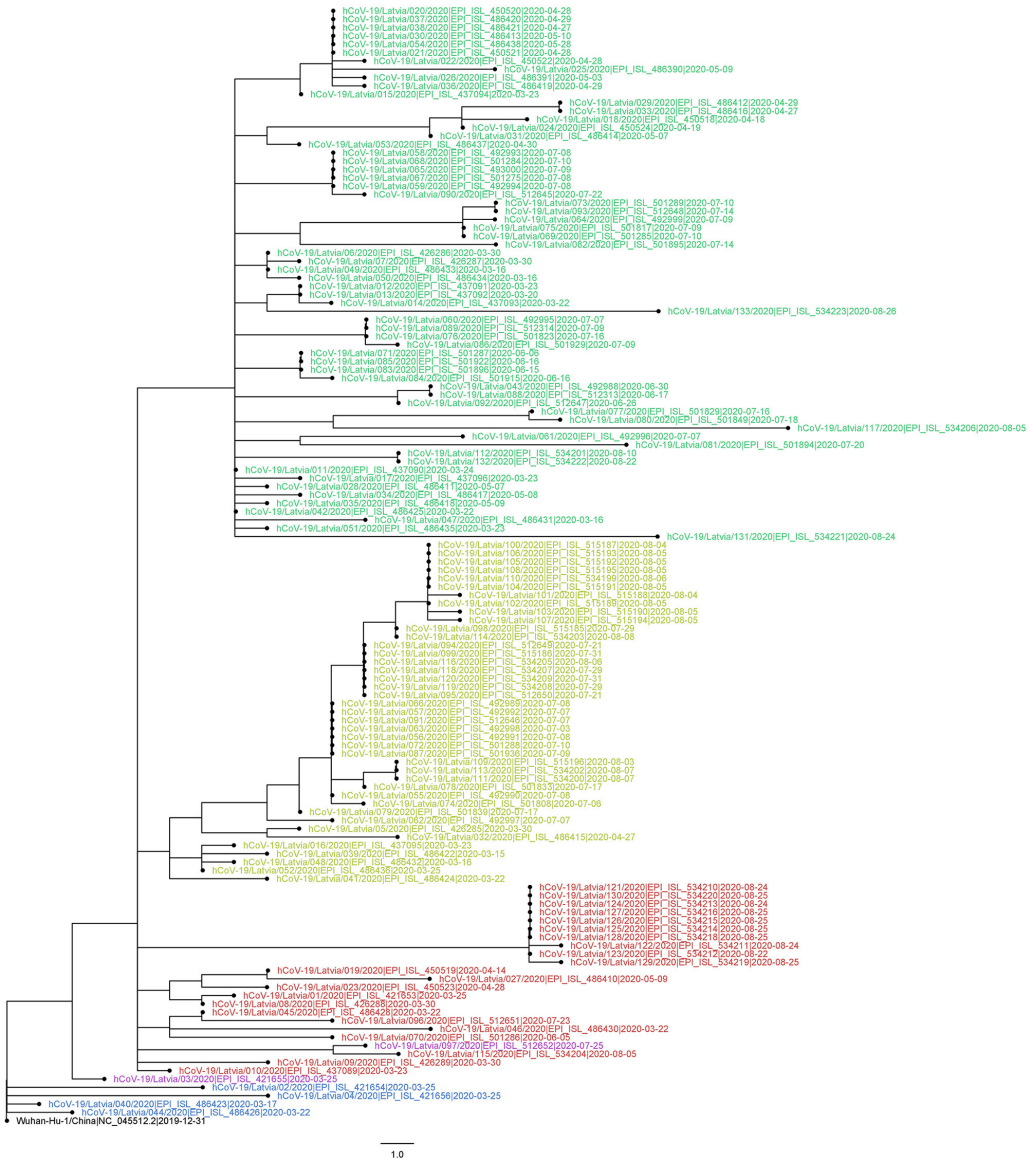
Evolutionary relationships of 133 sequenced Latvian and Wuhan-Hu-1 SARS-CoV-2 isolates. The evolutionary history was inferred using the Maximum-likelihood method allowing for polytomies. The tree is rooted at Wuhan-Hu-1 reference sequence. The tree is drawn to scale; branch lengths correspond to nucleotide substitutions. The analysis involved 134 nucleotide sequences (133 Latvian SARS-CoV-2 isolates and Wuhan-Hu-1 reference sequence). There were a total of 29,903 positions in the final dataset. Node labels are colored according to the GISAID major clade of particular isolate, as follows: green, GR; yellow, GH; red, G; blue, L; purple, O (other); black, Wuhan-Hu-1 reference sequence.

Maximum-likelihood phylogenetic tree was built to more apparently infer genetic distances between the samples (Figure 4). Although of satisfactory topology, supporting major clade clustering, the tree evidently shows the possible discrepancies between the reported sampling dates and expected sequence divergence (e.g., some of the samples most divergent from the root are dated with the end of April, while some of the most recently collected ones are notably less divergent), which, we believe, after manually inspecting the sequence alignment maps, is not attributable to sequencing errors or the possibility of coinfection by two different “strains.” Identical sequences sampled within a short date range (Figure 4) might be strongly indicative of epidemiologically linked transmission, given the relatively small daily amount of positive COVID-19 cases in Latvia that never exceeded 48, even during the “first wave” peaks of the disease spread up to late September, 2020.

While providing interesting insights on the COVID-19 situation in Latvia during the so-called “first coronavirus wave” (early March, 2020–mid-September, 2020), which might be representative of Baltics region to an extent, given the scarce amount of isolate genomes available from neighboring countries, it, however, should be noted, that the main drawback for each of the presented analyses is stemming from the available dataset—discrete early sampling with some of the dates since first positive case not being sampled at all (Figure 1). Another major drawback is the unavailability of complete patient/isolate epidemiological data that could be linked to the respective cases sequenced (e.g., sequence epidemiological linkage, patient travel history, etc.), which could be used to further refine the resolution of the analyses carried out, in the frame of this study. As currently Latvia is forced to be facing the “second coronavirus wave” that has not yet reached its peak, while the total number of cases in the country has more than tripled during month and a half since the middle of September, inclusion of additional data and retrospective sequencing of a larger number of cases that would allow for a more complete and in-depth analysis of the epidemiological situation throughout the whole epidemic in Latvia will be performed as soon as COVID-19 will cease to be a relevant threat and published elsewhere.

In conclusion, the high-throughput framework for SARS-CoV-2 isolate sequencing and data analysis in Latvia has been built by Latvian Biomedical Research and Study Center early on during the start of the pandemic, tested with the help of both, governmental and local private laboratory sample providers, and proposed as a pivotal tool to monitor the local outbreaks and aid in decision making. This framework has allowed us to ensure the successful sequencing of viral isolates from the majority of the new cases of epidemiological importance starting from the beginning of July, 2020 with fast date delivery to the Center for Disease Prevention and Control in Latvia allowing to link the epidemiological data with the genetic makeup of the priority isolates being sequenced and thus aiding the epidemiological investigations. We believe that this framework is of vital importance for rapid implementation of the most suitable public health measures, possible transmission history deduction, and viral evolution monitoring for the prevention of future epidemiological outbreaks and, with 14-day cumulative incidence reaching 2,202 as of 30th of October, 2020, is currently facing its hopefully greatest challenge up to date in the form of SARS-CoV-2 raging in Latvia with a whole new force.

## Data Availability Statement

The original contributions presented in the study are publicly available. This data can be found here: Each of the Latvian SARS-CoV-2 isolate complete genome sequences originating from Latvian Biomedical Research and Study centre underlying the study is available in GISAID EpiCoV coronavirus isolate sequence repository (https://www.gisaid.org) and the respective GISAID accession numbers of isolates are provided in Supplementary Table 3 associated with the article. NGS data associated with viral isolates described in this study (sequencing reads) have been deposited to European Nucleotide Archive (ENA) and are available under the following study accession: PRJEB40188 (https://www.ebi.ac.uk/ena/browser/view/PRJEB40188).

## Ethics Statement

The studies involving human participants were reviewed and approved by Central Medical Ethics Committee of Latvia (protocol No. 01-29.1/2429 and 01-29.1/1677). Written informed consent for participation was not required for this study in accordance with the national legislation and the institutional requirements.

## Author Contributions

JK, MU, VR, DF, ED, and UD conceptualized the study, acquired funding and approval of ethics committee, proposed and optimized the Latvian SARS-CoV-2 isolate sequencing framework, prioritized the cases to be sequenced, and supervised the study. LF, MP, AC, MG, DP, JS, and OS participated in patient testing and acquired the SARS-CoV-2 positive naso-/oropharyngeal swab samples, extracted hCoV-19 RNA and performed RT-PCR. LB, KM, LS, and LA prepared the NGS compatible libraries and performed the sequencing. IS, JP, and BV manipulated the raw sequencing data, generated isolate consensus sequences and performed variant calling. NZ, IS, and MU filtered out unsuccessfully sequenced isolates. NZ performed the phylogenetic analyses and prepared the tables/figures and prepared the final version of the manuscript. NZ, MU, IS, and JK wrote the draft version of the manuscript. All authors have interpreted results and edited the manuscript.

## Conflict of Interest

MP and AC were employed by the company Central Laboratory Ltd, Latvia. MG and DP were employed by the company E. Gulbja Laboratorija, Ltd, Latvia. The remaining authors declare that the research was conducted in the absence of any commercial or financial relationships that could be construed as a potential conflict of interest.

## Supplementary Material

Supplementary Figure 1 Methodological strategy plan for SARS-CoV-2 genome analysis based on different next-generation sequencing methods.

Supplementary Figure 2 Mutational landscape of Latvian SARS-CoV-2 isolates. *y*-axis shows the mutated position of a reference SARS-CoV-2 genome. *x*-axis shows the cumulative mutation count at a given position. Number to the right of bars indicate cumulative mutation count at a given position, and bars are color-coded according to the protein that the corresponding site participates in encoding. Note, that *y*-axis is discrete and only positions with mutations documented in local isolates are shown.

Supplementary Figure 3 Root-to-tip regression analysis of 133 Latvian SARS-CoV-2 isolates and Wuhan-Hu-1 sequence.

Supplementary Figure 4 Results of a phylogenetic signal inference in our dataset by likelihood mapping analysis as implemented in IQTREE v2.0.6. Overall quartet resolution (out of 134,000 quartets) was as follows: number of fully resolved quartets (regions 1 + 2 + 3): 86,669 (=64.68%); number of partly resolved quartets (regions 4 + 5 + 6): 3,454 (=2.58%); number of unresolved quartets (region 7): 43,877 (=32.74%).

Supplementary Table 1 Annotation and occurrence of 247 variants identifiable among 133 Latvian SARS-CoV-2 isolates sequenced (sorted by occurrence).

Supplementary Table 2 Latvian SARS-CoV-2 isolate sequencing approaches.

Supplementary Table 3 2019-nCoV RUO Kit (IDT) primers and probes for SARS-CoV-2 detection.

Supplementary Datasheet 1 GISAID SARS-CoV-2 sequence author acknowledgment table.
