# Supplementary figures and images for: First Report on the Latvian SARS-CoV-2 Isolate Genetic Diversity
Source: Front Med (Lausanne). 2021 Apr 6;8:626000. doi: 10.3389/fmed.2021.626000 (PMC8055824; doi:10.3389/fmed.2021.626000)

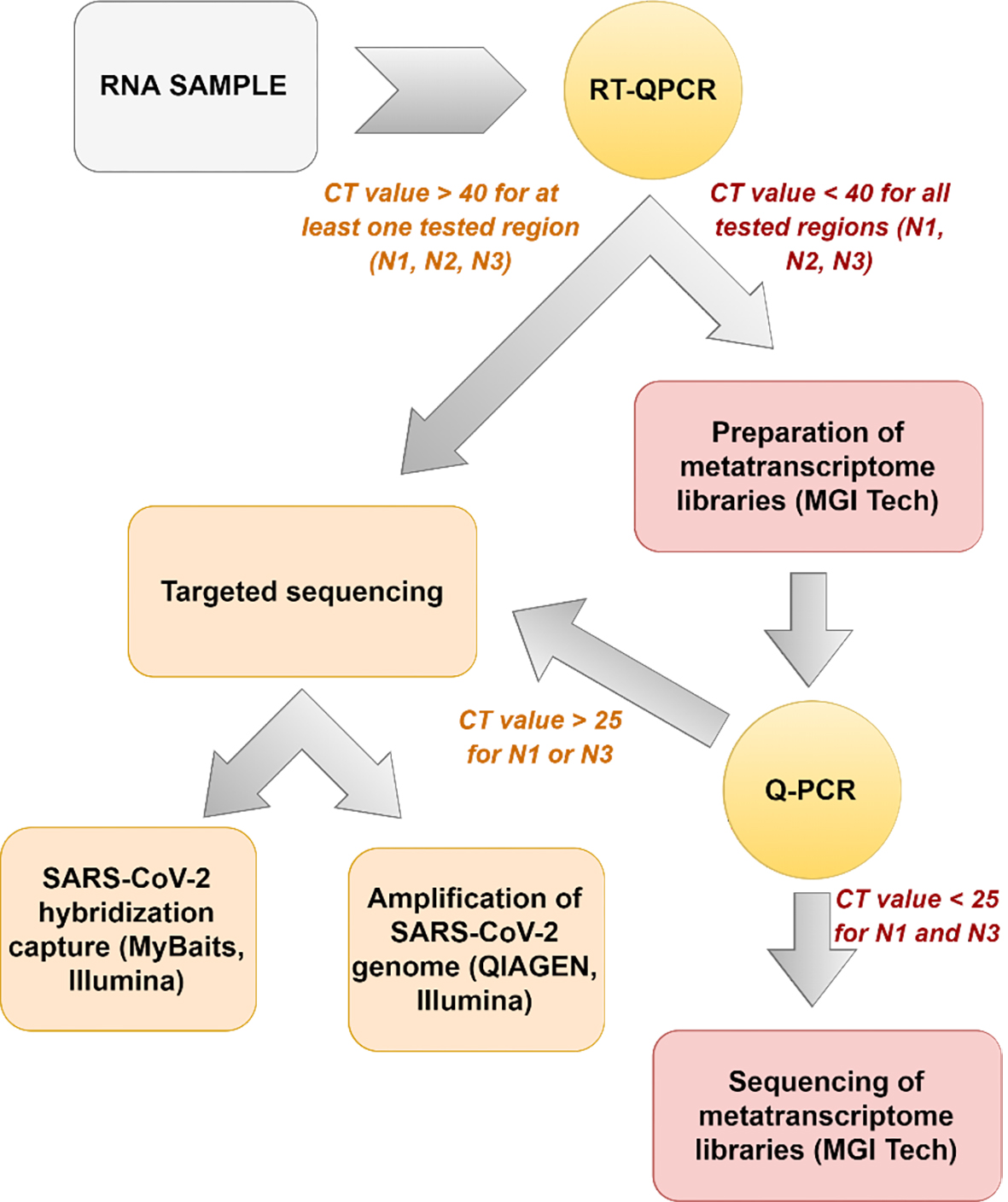

Supplement: Supplementary Figure 1 — Methodological strategy plan for SARS-CoV-2 genome analysis based on different next-generation sequencing methods. [file Image_1.JPEG]

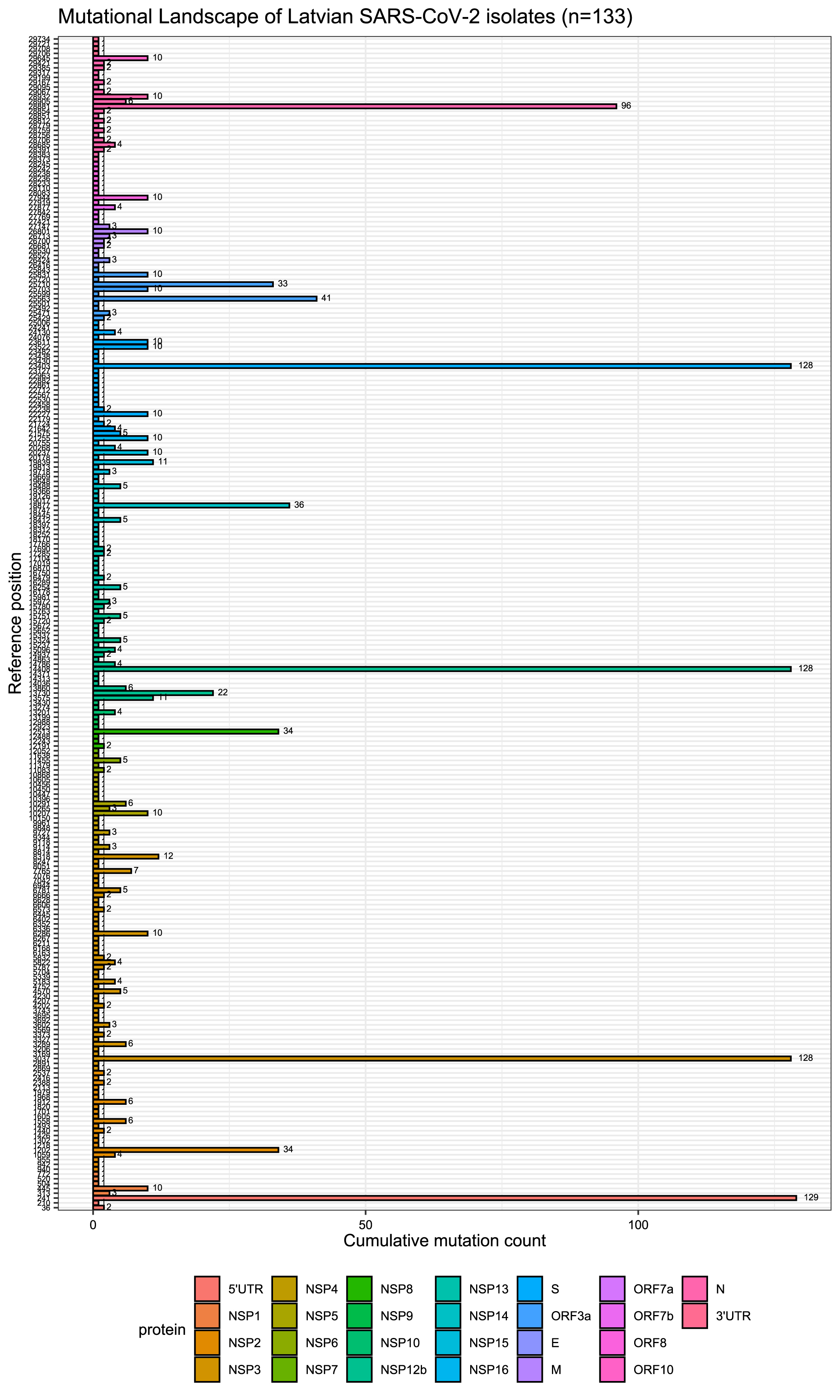

Supplement: Supplementary Figure 2 — Mutational landscape of Latvian SARS-CoV-2 isolates. y-axis shows the mutated position of a reference SARS-CoV-2 genome. x-axis shows the cumulative mutation count at a given position. Number to the right of bars indicate cumulative mutation count at a given position, and bars are color-coded according to the protein that the corresponding site participates in encoding. Note, that y-axis is discrete and only positions with mutations documented in local isolates are shown. [file Image_2.JPEG]

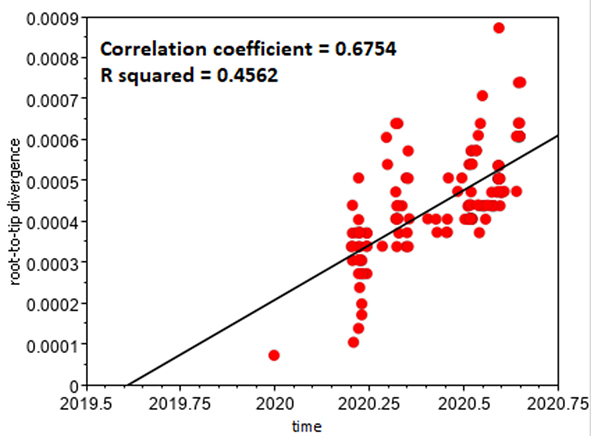

Supplement: Supplementary Figure 3 — Root-to-tip regression analysis of 133 Latvian SARS-CoV-2 isolates and Wuhan-Hu-1 sequence. [file Image_3.JPEG]

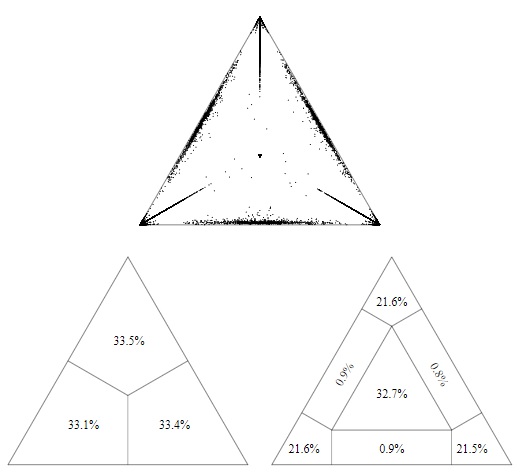

Supplement: Supplementary Figure 4 — Results of a phylogenetic signal inference in our dataset by likelihood mapping analysis as implemented in IQTREE v2.0.6. Overall quartet resolution (out of 134,000 quartets) was as follows: number of fully resolved quartets (regions 1 + 2 + 3): 86,669 (=64.68%); number of partly resolved quartets (regions 4 + 5 + 6): 3,454 (=2.58%); number of unresolved quartets (region 7): 43,877 (=32.74%). [file Image_4.JPEG]
