# Supplementary material for: First Report on the Latvian SARS-CoV-2 Isolate Genetic Diversity
Source: Front Med (Lausanne). 2021 Apr 6;8:626000. doi: 10.3389/fmed.2021.626000 (PMC8055824; doi:10.3389/fmed.2021.626000)
Supplement: Supplementary Table 3 — 2019-nCoV RUO Kit (IDT) primers and probes for SARS-CoV-2 detection. [file Table_3.docx]

Supplementary Table 3.

2019-nCoV RUO Kit (IDT) primers and probes for SARS-CoV-2 detection.

| **Name** | **Description** | **Oligonucleotide Sequence (5’>3’)** | **Label^1^** | **Working**  **Conc.** |
| --- | --- | --- | --- | --- |
| 2019-nCoV_N1-F | 2019-nCoV_N1  Forward Primer | 5’-GAC CCC AAA ATC AGC GAA AT-3’ | None | 20 µM |
| 2019-nCoV_N1-R | 2019-nCoV_N1  Reverse Primer | 5’-TCT GGT TAC TGC CAG TTG AAT CTG-3’ | None | 20 µM |
| 2019-nCoV_N1-P | 2019-nCoV_N1  Probe | 5’-FAM-ACC CCG CAT TAC GTT TGG TGG ACC-BHQ1-3’ | FAM, BHQ-1 | 5 µM |
| 2019-nCoV_N2-F | 2019-nCoV_N2  Forward Primer | 5’-TTA CAA ACA TTG GCC GCA AA-3’ | None | 20 µM |
| 2019-nCoV_N2-R | 2019-nCoV_N2  Reverse Primer | 5’-GCG CGA CAT TCC GAA GAA-3’ | None | 20 µM |
| 2019-nCoV_N2-P | 2019-nCoV_N2  Probe | 5’-FAM-ACA ATT TGC CCC CAG CGC TTC AG-BHQ1-3’ | FAM, BHQ-1 | 5 µM |
| 2019-nCoV_N3-F | 2019-nCoV_N3  Forward Primer | 5’-GGG AGC CTT GAA TAC ACC AAA A-3’ | None | 20 µM |
| 2019-nCoV_N3-R | 2019-nCoV_N3  Reverse Primer | 5’-TGT AGC ACG ATT GCA GCA TTG-3’ | None | 20 µM |
| 2019-nCoV_N3-P | 2019-nCoV_N3  Probe | 5’-FAM-AYC ACA TTG GCA CCC GCA ATC CTG-BHQ1-3’ | FAM, BHQ-1 | 5 µM |
| RP-F | RNAse P Forward  Primer | 5’-AGA TTT GGA CCT GCG AGC G-3’ | None | 20 µM |
| RP-R | RNAse P Reverse  Primer | 5’-GAG CGG CTG TCT CCA CAA GT-3’ | None | 20 µM |
| RP-P | RNAse P  Probe | 5’-FAM – TTC TGA CCT GAA GGC TCT GCG CG – BHQ-1-3’ | FAM, BHQ-1 | 5 µM |

## ^1^TaqMan® probes are labeled at the 5'-end with the reporter molecule 6-carboxyfluorescein (FAM) and with the quencher, Black Hole Quencher 1 (BHQ-1) (Biosearch Technologies, Inc., Novato, CA) at the 3'- end. The sequences of primers and probes are also published in the webpage of Centers of Disease Control and Prevention (https://www.cdc.gov/).
