# Supplementary material for: First Report on the Latvian SARS-CoV-2 Isolate Genetic Diversity
Source: Front Med (Lausanne). 2021 Apr 6;8:626000. doi: 10.3389/fmed.2021.626000 (PMC8055824; doi:10.3389/fmed.2021.626000)
Supplement: Supplementary Datasheet 1 — GISAID SARS-CoV-2 sequence author acknowledgment table. [file Data_Sheet_1.PDF]

We gratefully acknowledge the following Authors from the Originating laboratories responsible for obtaining the specimens, as well as the Submitting laboratories where the genome data were generated and shared via GISAID, on which this research is based.

All Submitters of data may be contacted directly via www.gisaid.org

Accession ID	Originating Laboratory	Submitting Laboratory	Authors
EPI_ISL_421653, EPI_ISL_421654, EPI_ISL_421655, EPI_ISL_421656, EPI_ISL_426285, EPI_ISL_426286, EPI_ISL_426287, EPI_ISL_426288, EPI_ISL_426289	E. Gulbja Laboratorija	Latvian Biomedical Research and Study Centre	Ivars Silamiķelis, Kaspars Megnis, Monta Ustinova, Ņikita Zrelovs, Vita Rovīte, Mikus Gavars, Dmitrijs Perminovs, Uga Dumpis, Jānis Kloviņš
EPI_ISL_437089, EPI_ISL_437090, EPI_ISL_437091, EPI_ISL_437092, EPI_ISL_437093, EPI_ISL_437094, EPI_ISL_437095, EPI_ISL_437096	Latvijas Infektoloģijas centrs	Latvian Biomedical Research and Study Centre	Ivars Silamiķelis, Kaspars Megnis, Monta Ustinova, Ņikita Zrelovs, Vita Rovīte, Jeļena Storoženko, Tatjana Kolupajeva, Oksana Savicka, Uga Dumpis, Jānis Kloviņš
EPI_ISL_450518, EPI_ISL_450519	E. Gulbja Laboratorija	Latvian Biomedical Research and Study Centre	Ivars Silamiķelis, Kaspars Megnis, Monta Ustinova, Ņikita Zrelovs, Vita Rovīte, Mikus Gavars, Dmitrijs Perminovs, Uga Dumpis, Jānis Kloviņš
EPI_ISL_450520, EPI_ISL_450521, EPI_ISL_450522, EPI_ISL_450523, EPI_ISL_450524	Centrālā Laboratorija	Latvian Biomedical Research and Study Centre	Ivars Silamiķelis, Kaspars Megnis, Monta Ustinova, Ņikita Zrelovs, Vita Rovīte, Stella Lapiņa, Jana Osīte, Marta Priedīte, Uga Dumpis, Jānis Kloviņš
EPI_ISL_486390, EPI_ISL_486391	Centrālā laboratorija	Latvian Biomedical Research and Study Centre	Ivars Silamiķelis, Kaspars Megnis, Monta Ustinova, Ņikita Zrelovs, Vita Rovīte, Stella Lapiņa, Jana Osīte, Marta Priedīte, Uga Dumpis, Jānis Kloviņš
EPI_ISL_486410	Centrala laboratorija	Latvian Biomedical Research and Study Centre	Ivars Silamiķelis, Kaspars Megnis, Monta Ustinova, Ņikita Zrelovs, Vita Rovīte, Stella Lapiņa, Jana Osīte, Marta Priedīte, Uga Dumpis, Jānis Kloviņš
EPI_ISL_486411	Centrālā laboratorija	Latvian Biomedical Research and Study Centre	Ivars Silamiķelis, Kaspars Megnis, Monta Ustinova, Ņikita Zrelovs, Vita Rovīte, Stella Lapiņa, Jana Osīte, Marta Priedīte, Uga Dumpis, Jānis Kloviņš
EPI_ISL_486412	Centrālā laboratorija	Latvian Biomedical Research and Study Centre	Ivars Silamiķelis, Kaspars Megnis, Monta Ustinova, Ņikita Zrelovs, Vita Rovīte, Stella Lapiņa, Jana Osīte, Marta Priedīte, Uga Dumpis, Jānis Kloviņš
EPI_ISL_486413, EPI_ISL_486414	Centrālā laboratorija	Latvian Biomedical Research and Study Centre	Ivars Silamiķelis, Kaspars Megnis, Monta Ustinova, Ņikita Zrelovs, Vita Rovīte, Stella Lapiņa, Jana Osīte, Marta Priedīte, Uga Dumpis, Jānis Kloviņš
EPI_ISL_486415	Centrālā laboratorija	Latvian Biomedical Research and Study Centre	Ivars Silamiķelis, Kaspars Megnis, Monta Ustinova, Ņikita Zrelovs, Vita Rovīte, Stella Lapiņa, Jana Osīte, Marta Priedīte, Uga Dumpis, Jānis Kloviņš
EPI_ISL_486416	Centrālā laboratorija	Latvian Biomedical Research and Study Centre	Ivars Silamiķelis, Kaspars Megnis, Monta Ustinova, Ņikita Zrelovs, Vita Rovīte, Stella Lapiņa, Jana Osīte, Marta Priedīte, Uga Dumpis, Jānis Kloviņš
EPI_ISL_486417	Centrala laboratorija	Latvian Biomedical Research and Study Centre	Ivars Silamiķelis, Kaspars Megnis, Monta Ustinova, Ņikita Zrelovs, Vita Rovīte, Stella Lapiņa, Jana Osīte, Marta Priedīte, Uga Dumpis, Jānis Kloviņš
EPI_ISL_486418	Centrālā laboratorija	Latvian Biomedical Research and Study Centre	Ivars Silamiķelis, Kaspars Megnis, Monta Ustinova, Ņikita Zrelovs, Vita Rovīte, Stella Lapiņa, Jana Osīte, Marta Priedīte, Uga Dumpis, Jānis Kloviņš
EPI_ISL_486419	Centrālā laboratorija	Latvian Biomedical Research and Study Centre	Ivars Silamiķelis, Kaspars Megnis, Monta Ustinova, Ņikita Zrelovs, Vita Rovīte, Stella Lapiņa, Jana Osīte, Marta Priedīte, Uga Dumpis, Jānis Kloviņš
EPI_ISL_486420, EPI_ISL_486421	Centrālā laboratorija	Latvian Biomedical Research and Study Centre	Ivars Silamiķelis, Kaspars Megnis, Monta Ustinova, Ņikita Zrelovs, Vita Rovīte, Stella Lapiņa, Jana Osīte, Marta Priedīte, Uga Dumpis, Jānis Kloviņš
EPI_ISL_486422, EPI_ISL_486423, EPI_ISL_486424, EPI_ISL_486425, EPI_ISL_486426, EPI_ISL_486428, EPI_ISL_486430, EPI_ISL_486431, EPI_ISL_486432, EPI_ISL_486433, EPI_ISL_486434, EPI_ISL_486435			
see above	Latvijas Infektoloģijas centrs	Latvian Biomedical Research and Study Centre	Ivars Silamiķelis, Kaspars Megnis, Monta Ustinova, Ņikita Zrelovs, Vita Rovīte, Jeļena Storoženko, Tatjana Kolupajeva, Oksana Savicka, Uga Dumpis, Jānis Kloviņš
EPI_ISL_486436	Latvijas Infektoloģijas centrs	Latvian Biomedical Research and Study Centre	Ivars Silamiķelis, Kaspars Megnis, Monta Ustinova, Ņikita Zrelovs, Vita Rovīte, Jeļena Storoženko, Tatjana Kolupajeva, Oksana Savicka, Uga Dumpis, Jānis Kloviņš
EPI_ISL_486437	Centrālā laboratorija	Latvian Biomedical Research and Study Centre	Ivars Silamiķelis, Kaspars Megnis, Monta Ustinova, Ņikita Zrelovs, Vita Rovīte, Stella Lapiņa, Jana Osīte, Marta Priedīte, Uga Dumpis, Jānis Kloviņš
EPI_ISL_486438	E. Gulbja laboratorija	Latvian Biomedical Research and Study Centre	Ivars Silamiķelis, Kaspars Megnis, Monta Ustinova, Ņikita Zrelovs, Vita Rovīte, Mikus Gavars, Dmitrijs Perminovs, Uga Dumpis, Jānis Kloviņš
EPI_ISL_492988, EPI_ISL_492989	Centrālā laboratorija	Latvian Biomedical Research and Study Centre	Ivars Silamiķelis, Kaspars Megnis, Monta Ustinova, Ņikita Zrelovs, Vita Rovīte, Stella Lapiņa, Jana Osīte, Marta Priedīte, Uga Dumpis, Jānis Kloviņš
EPI_ISL_492990	Centrālā laboratorija	Latvian Biomedical Research and Study Centre	Ivars Silamiķelis, Kaspars Megnis, Monta Ustinova, Ņikita Zrelovs, Vita Rovīte, Stella Lapiņa, Jana Osīte, Marta Priedīte, Uga Dumpis, Jānis Kloviņš
EPI_ISL_492991, EPI_ISL_492992	Centrālā laboratorija	Latvian Biomedical Research and Study Centre	Ivars Silamiķelis, Kaspars Megnis, Monta Ustinova, Ņikita Zrelovs, Vita Rovīte, Stella Lapiņa, Jana Osīte, Marta Priedīte, Uga Dumpis, Jānis Kloviņš
EPI_ISL_492993, EPI_ISL_492994, EPI_ISL_492995, EPI_ISL_492996, EPI_ISL_492997, EPI_ISL_492998, EPI_ISL_492999, EPI_ISL_493000	E. Gulbja Laboratorija	Latvian Biomedical Research and Study Centre	Ivars Silamiķelis, Kaspars Megnis, Monta Ustinova, Ņikita Zrelovs, Vita Rovīte, Mikus Gavars, Dmitrijs Perminovs, Uga Dumpis, Jānis Kloviņš
EPI_ISL_501275	E. Gulbja Laboratorija	Latvian Biomedical Research and Study Centre	Ivars Silamiķelis, Kaspars Megnis, Monta Ustinova, Ņikita Zrelovs, Vita Rovīte, Mikus Gavars, Dmitrijs Perminovs, Uga Dumpis, Jānis Kloviņš
EPI_ISL_501284, EPI_ISL_501285	E. Gulbja Laboratorija	Latvian Biomedical Research and Study Centre	Ivars Silamiķelis, Kaspars Megnis, Monta Ustinova, Ņikita Zrelovs, Vita Rovīte, Mikus Gavars, Dmitrijs Perminovs, Uga Dumpis, Jānis Kloviņš
EPI_ISL_501286, EPI_ISL_501287, EPI_ISL_501288, EPI_ISL_501289, EPI_ISL_501808, EPI_ISL_501817	Centrālā laboratorija	Latvian Biomedical Research and Study Centre	Ivars Silamiķelis, Kaspars Megnis, Monta Ustinova, Ņikita Zrelovs, Vita Rovīte, Stella Lapiņa, Jana Osīte, Marta Priedīte, Uga Dumpis, Jānis Kloviņš
EPI_ISL_501823, EPI_ISL_501829, EPI_ISL_501833, EPI_ISL_501839, EPI_ISL_501849, EPI_ISL_501894	E. Gulbja Laboratorija	Latvian Biomedical Research and Study Centre	Ivars Silamiķelis, Kaspars Megnis, Monta Ustinova, Ņikita Zrelovs, Vita Rovīte, Mikus Gavars, Dmitrijs Perminovs, Uga Dumpis, Jānis Kloviņš
EPI_ISL_501895, EPI_ISL_501896, EPI_ISL_501915, EPI_ISL_501922	Centrālā laboratorija	Latvian Biomedical Research and Study Centre	Ivars Silamiķelis, Kaspars Megnis, Monta Ustinova, Ņikita Zrelovs, Vita Rovīte, Stella Lapiņa, Jana Osīte, Marta Priedīte, Uga Dumpis, Jānis Kloviņš
EPI_ISL_501929, EPI_ISL_501936, EPI_ISL_512313, EPI_ISL_512314, EPI_ISL_512645, EPI_ISL_512646	E. Gulbja Laboratorija	Latvian Biomedical Research and Study Centre	Ivars Silamiķelis, Kaspars Megnis, Monta Ustinova, Ņikita Zrelovs, Vita Rovīte, Mikus Gavars, Dmitrijs Perminovs, Uga Dumpis, Jānis Kloviņš
EPI_ISL_512647, EPI_ISL_512648	Latvijas Infektoloģijas centrs	Latvian Biomedical Research and Study Centre	Ivars Silamiķelis, Kaspars Megnis, Monta Ustinova, Ņikita Zrelovs, Vita Rovīte, Jeļena Storoženko, Tatjana Kolupajeva, Oksana Savicka, Uga Dumpis, Jānis Kloviņš
EPI_ISL_512649	Latvijas Infektoloģijas centrs	Latvian Biomedical Research and Study Centre	Ivars Silamiķelis, Kaspars Megnis, Monta Ustinova, Ņikita Zrelovs, Vita Rovīte, Jeļena Storoženko, Tatjana Kolupajeva, Oksana Savicka, Uga Dumpis, Jānis Kloviņš
EPI_ISL_512650, EPI_ISL_512651, EPI_ISL_512652, EPI_ISL_515185, EPI_ISL_515186	Latvijas Infektoloģijas centrs	Latvian Biomedical Research and Study Centre	Ivars Silamiķelis, Kaspars Megnis, Monta Ustinova, Ņikita Zrelovs, Vita Rovīte, Jeļena Storoženko, Tatjana Kolupajeva, Oksana Savicka, Uga Dumpis, Jānis Kloviņš
EPI_ISL_515187, EPI_ISL_515188, EPI_ISL_515189, EPI_ISL_515190, EPI_ISL_515191, EPI_ISL_515192, EPI_ISL_515193, EPI_ISL_515194, EPI_ISL_515195	E. Gulbja Laboratorija	Latvian Biomedical Research and Study Centre	Ivars Silamiķelis, Kaspars Megnis, Monta Ustinova, Ņikita Zrelovs, Vita Rovīte, Mikus Gavars, Dmitrijs Perminovs, Uga Dumpis, Jānis Kloviņš
EPI_ISL_515196	Centrālā laboratorija	Latvian Biomedical Research and Study Centre	Ivars Silamiķelis, Kaspars Megnis, Monta Ustinova, Ņikita Zrelovs, Vita Rovīte, Stella Lapiņa, Jana Osīte, Marta Priedīte, Uga Dumpis, Jānis Kloviņš
EPI_ISL_534199	E. Gulbja Laboratorija	Latvian Biomedical Research and Study Centre	Ivars Silamiķelis, Jānis Pjalkovskis, Kaspars Megnis, Monta Ustinova, Ņikita Zrelovs, Vita Rovīte, Mikus Gavars, Dmitrijs Perminovs, Uga Dumpis, Jānis Kloviņš
EPI_ISL_534200, EPI_ISL_534201, EPI_ISL_534202	Centrālā laboratorija	Latvian Biomedical Research and Study Centre	Ivars Silamiķelis, Jānis Pjalkovskis, Kaspars Megnis, Monta Ustinova, Ņikita Zrelovs, Vita Rovīte, Stella Lapiņa, Jana Osīte, Marta Priedīte, Uga Dumpis, Jānis Kloviņš
EPI_ISL_534203	E. Gulbja Laboratorija	Latvian Biomedical Research and Study Centre	Ivars Silamiķelis, Jānis Pjalkovskis, Kaspars Megnis, Monta Ustinova, Ņikita Zrelovs, Vita Rovīte, Mikus Gavars, Dmitrijs Perminovs, Uga Dumpis, Jānis Kloviņš
EPI_ISL_534204, EPI_ISL_534205	Latvijas Infektoloģijas centrs	Latvian Biomedical Research and Study Centre	Ivars Silamiķelis, Jānis Pjalkovskis, Kaspars Megnis, Monta Ustinova, Ņikita Zrelovs, Vita Rovīte, Jeļena Storoženko, Tatjana Kolupajeva, Oksana Savicka, Uga Dumpis, Jānis Kloviņš
EPI_ISL_534206, EPI_ISL_534207, EPI_ISL_534208	Centrālā laboratorija	Latvian Biomedical Research and Study Centre	Ivars Silamiķelis, Jānis Pjalkovskis, Kaspars Megnis, Monta Ustinova, Ņikita Zrelovs, Vita Rovīte, Stella Lapiņa, Jana Osīte, Marta Priedīte, Uga Dumpis, Jānis Kloviņš
EPI_ISL_534209	Latvijas Infektoloģijas centrs	Latvian Biomedical Research and Study Centre	Ivars Silamiķelis, Jānis Pjalkovskis, Kaspars Megnis, Monta Ustinova, Ņikita Zrelovs, Vita Rovīte, Jeļena Storoženko, Tatjana Kolupajeva, Oksana Savicka, Uga Dumpis, Jānis Kloviņš
EPI_ISL_534210, EPI_ISL_534211	Centrālā laboratorija	Latvian Biomedical Research and Study Centre	Ivars Silamiķelis, Jānis Pjalkovskis, Kaspars Megnis, Monta Ustinova, Ņikita Zrelovs, Vita Rovīte, Stella Lapiņa, Jana Osīte, Marta Priedīte, Uga Dumpis, Jānis Kloviņš
EPI_ISL_534212, EPI_ISL_534213, EPI_ISL_534214, EPI_ISL_534215, EPI_ISL_534216, EPI_ISL_534218, EPI_ISL_534219	E. Gulbja Laboratorija	Latvian Biomedical Research and Study Centre	Ivars Silamiķelis, Jānis Pjalkovskis, Kaspars Megnis, Monta Ustinova, Ņikita Zrelovs, Vita Rovīte, Mikus Gavars, Dmitrijs Perminovs, Uga Dumpis, Jānis Kloviņš
EPI_ISL_534220	Centrālā laboratorija	Latvian Biomedical Research and Study Centre	Ivars Silamiķelis, Jānis Pjalkovskis, Kaspars Megnis, Monta Ustinova, Ņikita Zrelovs, Vita Rovīte, Stella Lapiņa, Jana Osīte, Marta Priedīte, Uga Dumpis, Jānis Kloviņš
EPI_ISL_534221	E. Gulbja Laboratorija	Latvian Biomedical Research and Study Centre	Ivars Silamiķelis, Jānis Pjalkovskis, Kaspars Megnis, Monta Ustinova, Ņikita Zrelovs, Vita Rovīte, Mikus Gavars, Dmitrijs Perminovs, Uga Dumpis, Jānis Kloviņš
EPI_ISL_534222, EPI_ISL_534223	Centrālā laboratorija	Latvian Biomedical Research and Study Centre	Ivars Silamiķelis, Jānis Pjalkovskis, Kaspars Megnis, Monta Ustinova, Ņikita Zrelovs, Vita Rovīte, Stella Lapiņa, Jana Osīte, Marta Priedīte, Uga Dumpis, Jānis Kloviņš
